# Police officers' prejudice and distrust towards racialized groups is related to internal motivation to suppress prejudice and negative intergroup contact

**DOI:** 10.1111/bjso.70094

**Published:** 2026-05-15

**Authors:** Marleen Stelter, Iniobong Essien, Jan Malte von Bargen, Oliver Christ

**Affiliations:** ^1^ FernUniversität in Hagen Hagen Germany; ^2^ Leuphana Universität Lüneburg Lüneburg Germany; ^3^ Universität Freiburg Freiburg Germany

**Keywords:** discrimination, intergroup contact, police bias, prejudice, shooter bias, stereotypes

## Abstract

Racialized individuals experience different interactions with the police compared to non‐racialized individuals. This study investigates biases among German police officers (*N* = 208) towards individuals perceived as Arab. Police officers demonstrated shooter biases in a first‐person shooter task, rated Arab individuals as less trustworthy, and expressed a preference for White individuals over Arab individuals. These biases closely mirrored those found in a civilian convenience sample (*N* = 237), with one notable difference: police officers showed significantly greater distrust towards Arab individuals than civilians. This heightened distrust was mediated by social dominance orientation and negative perceptions of intergroup contact. Additionally, internal motivation to suppress prejudice was as a strong predictor of both group preference and distrust in both samples. Collectively, these findings highlight that police officers, as a reflection of society, exhibit pervasive biases towards racialized groups, which might impact their interactions with minoritized communities.

## INTRODUCTION

Are police officers biased against racialized groups? A growing body of research suggests that racialized individuals experience policing differently than their non‐racialized counterparts. For example, Black citizens in the United States are disproportionately stopped by police (Stelter et al., 2022) and treated with less respect than White citizens (Voigt et al., 2017). Such disparities in policing outcomes may reflect both institutional practices and individual officers' biases (e.g. Camp, 2024; Epp et al., 2017). While institutional factors such as policies, training and organizational culture undoubtedly shape policing outcomes, this study focuses on the role of individual biases among officers, because they represent the psychological mechanisms through which broader systemic inequalities are enacted and perpetuated in everyday policing.

### Individual biases matter in policing

Individual biases are particularly consequential in policing due to the high degree of discretion officers have in interpreting and responding to situations. Officers' decisions are not solely guided by formal protocols but are also shaped by personal beliefs, perceptions and motivations (Swencionis & Goff, 2017). From a psychological perspective, such biases can influence perceptual and decision‐making processes, especially under conditions of stress and ambiguity—contexts that are common in police work (Correll, Park, Judd, & Wittenbrink, 2007; Cox & Devine, 2019; Fazio, 1990; Hugenberg & Bodenhausen, 2003). Under these conditions, officers are more likely to rely on heuristic processing and stereotype activation, increasing the likelihood of biased judgements and behaviours.

The societal implications of police officers' biases are profound. Police officers possess a monopoly on the (legitimate) use of force and play a central role in shaping public perceptions of law enforcement institutions (Camp, 2024). When officers are perceived as biased, they signal institutional distrust towards marginalized communities, undermining perceptions of procedural justice and legitimacy (Camp et al., 2021; Espín Grau & Klaus, 2022; Kauff et al., 2017; Tyler et al., 2015). These dynamics underscore the need for systematic investigations into the psychological underpinnings of police bias.

#### Forms of bias in policing

Police officers' biases can manifest in multiple, interrelated forms, including biased behaviour, stereotypes, prejudice and more specific intergroup emotions such as distrust. Behavioural biases refer to discriminatory actions—such as disproportionate stops, use of force or differential treatment—that may arise from underlying prejudice or stereotypes. One example of behavioural bias relevant to police work is shooter bias (Correll et al., 2002), whereby officers are more likely to mistakenly perceive racialized individuals, particularly Black or Arab men, as armed or dangerous, leading to unjustified use of force. Experimental studies have documented shooter bias among police officers across several contexts (Akinola & Mendes, 2012; Correll, Park, Judd, & Wittenbrink, Sadler, & Keese, 2007; Lima et al., 2018; Sadler et al., 2012; Stelter et al., 2023). However, other work reports more mixed findings: some studies report bias only in early task trials (Plant & Peruche, 2005), others find no bias (Andersen et al., 2023; Cox et al., 2014) and some even report reversed biases favouring Black targets (James et al., 2016). Evidence is similarly mixed regarding whether officers show larger, similar or smaller shooter biases than civilians (Sim et al., 2013; Correll, Park, Judd, Wittenbrink, Sadler, & Keesee, 2007, Studies 1–2; Lima et al., 2018; Stelter et al., 2023). These inconsistencies highlight the ongoing need for systematic behavioural research with active‐duty officers.

Stereotypes in policing can take the form of threat‐ and crime‐related associations, linking racialized groups to danger, aggression or criminality (Correll, Park, Judd, Wittenbrink, Sadler, & Keesee, 2007; Hall et al., 2016). Such cognitive biases can influence perception and decision‐making, especially under stress or ambiguity (Correll et al., 2015). Research shows that racialized individuals, particularly Black and Arab men, are more readily perceived as threatening or armed (Correll, Park, Judd, Wittenbrink, Sadler, & Keesee, 2007; Hall et al., 2016), and that such stereotypes distort police officers' split‐second judgements in high‐pressure situations (Correll et al., 2015).

Prejudice reflects negative evaluations or affective reactions towards racialized groups. In policing, such evaluative biases can shape how officers perceive and evaluate individuals during encounters (Carvalho et al., 2021). For example, an officer who holds more negative prejudice may interpret the behaviour of a racialized individual as more hostile or deceptive than it actually is. Survey research across several countries, including Germany, the United States, Australia and Finland, indicates that police officers often report higher levels of prejudice towards racialized minorities than recruits, civilians, teachers or social workers (Colman & Gorman, 1982; Deutsche Hochschule der Polizei, 2024; Gatto et al., 2010; Kemme et al., 2020; Wortley & Homel, 1995; Pitkänen & Kouki, 2002; but see Clasen et al., 2024). However, it should be noted that these studies rely mostly on self‐report measures, which capture only limited dimensions of bias and may be susceptible to social desirability concerns, potentially biasing police officers' responses to adhere to local norms.

Beyond these behavioural, cognitive and evaluative biases, an important intergroup emotion in the policing context may be distrust, characterized by suspicion and the expectation of deceit (Hutchings et al., 2024). In policing, distrust may play a critical role in racial profiling, where vague notions of ‘suspicious behaviour’ can justify disproportionate scrutiny (Epp et al., 2017; Minhas & Walsh, 2021). Recent work suggests that distrust may be a distinct and powerful driver of biased policing, yet it remains underexplored in empirical research (Hutchings et al., 2024). Understanding distrust is particularly important because it may operate independently of the endorsement of prejudice or stereotypes and still influence officers' decisions.

Across these different forms of intergroup bias in policing, the empirical literature reveals important gaps. Empirical evidence on stereotypes and prejudice is more established but relies largely on self‐reports. Behavioural biases have been documented across a number of studies, although other work shows that these effects may vary across contexts and task designs. Lastly, intergroup emotions such as distrust remain largely unexamined in the context of policing. Together, these gaps underscore the need of systematic research on the prevalence and psychological underpinnings of police bias.

### Psychological processes underlying police officer bias

In addition to documenting bias, focusing on individual police officers allows examining psychological processes that underlie biased policing. One important ideological factor is social dominance orientation, which reflects a preference for maintaining hierarchical relations between groups (Sidanius & Pratto, 1999). Social dominance orientation predicts prejudice and is also associated with attraction to hierarchy‐enhancing institutions such as the police (Haley & Sidanius, 2005; Sidanius et al., 1994). Officers high in social dominance orientation may also be more likely to interpret intergroup encounters through a lens of threat and control, reinforcing biased perceptions and behaviours (Swencionis et al., 2021). Thus, social dominance orientation may serve as both a selection mechanism and a psychological mechanism that reinforces bias among police officers.

In addition to ideological orientations, police officers' personal motivations to suppress prejudice may play an important role. Internal motivation to suppress prejudice reflects the extent to which individuals are personally committed to egalitarian values (Plant & Devine, 1998). Police officers with lower internal motivation to suppress prejudice may be more likely to act on biases. Research indicates that motivation to suppress prejudice is initially similar between police recruits and civilians but declines with on‐the‐job experience (Gatto et al., 2010). Police officers' motivations to suppress prejudice may thus not be different from civilians per se, but with increasing on‐the‐job experience, police officers may be more inclined to openly express and act upon prejudice. This finding points to potential socialization effects of on‐the‐job experiences on internal motivation to suppress prejudice.

Indeed, the normative climate of police organizations may play a critical role in shaping police officers' attitudes. Police forces, as hierarchy‐enhancing institutions, may influence police officers' attitudes over time. Scholars point to distinct ‘cop cultures’ that emphasize loyalty and solidarity, fostering an ‘us vs. them’ mentality which can extend beyond criminal suspects to encompass entire marginalized communities (e.g. Bowling et al., 2019). This cultural framing may reduce external pressures to suppress prejudice and normalize biased behaviour, thereby reinforcing the psychological mechanisms described above.

Moreover, police officers' biases towards racialized groups may also be shaped by how they construe intergroup contact experiences in their daily work. Routine encounters with racialized individuals in contexts associated with crime and poverty (Fassin, 2013) may reinforce threat‐related schemas and stereotype‐consistent interpretations (Smith & Alpert, 2007). These experiences can create a feedback loop: negative contact increases prejudice (Barlow et al., 2012), which may in turn lead to disrespectful behaviour (Voigt et al., 2017), potentially escalating situations and causing confrontational encounters (Todak & James, 2018). Repeated negative interactions can further deepen distrust and prejudice towards racialized communities (Wortley & Homel, 1995).

Lastly, previous research has raised the question whether bias towards racialized groups is widespread among police officers or if it is confined to a few individuals (‘a few bad apples’; Chalfin & Kaplan, 2021). If bias is confined to a few police officers, solutions could focus on identifying, training or removing these individuals from decision‐making roles. However, if bias is widespread, addressing police bias may require systemic policy changes. Recent analyses have demonstrated that discriminatory judicial decisions and discrimination in the job and housing markets are prevalent across the population and not confined to a small set of individuals (Galvan et al., 2026). Whether bias among police officers is widespread or concentrated among a few biased individuals remains an open question.

Based on these considerations, this study examines how individual‐level factors, including ideological beliefs, personal motivations, workplace norms and everyday experiences with racialized communities, contribute to police officers' biases towards racialized groups. While these (non‐exhaustive) factors are often discussed in theory, there is a lack of empirical research that systematically investigates them in real‐world policing contexts. Existing studies are limited in scope, often relying on small samples or narrow methods. This study addresses these gaps by using a multi‐method approach to provide a more comprehensive understanding of the underlying processes of bias among police officers.

## THE PRESENT RESEARCH

The present research examines whether police officers exhibit biases towards individuals perceived as Arab or Muslim,^1^ racialized groups that are highly stigmatized in many European countries (e.g. Strabac & Listhaug, 2008). We examine police bias towards this group in the German context. People with Arab or Muslim backgrounds constitute a substantial proportion of the population in Germany, reflecting immigration from the Middle East, North Africa and Turkey over several decades (Islam in Germany: Facts and figures, 2025). Public debates often link Arab and Muslim communities to issues of security, perpetuating threat‐related stereotypes (Stürmer et al., 2019). Because people perceived as Arab or Muslim make up a large population in Germany, police officer bias against them may affect institutional trust and the everyday experiences of millions (Evangelist, 2022). At the same time, empirical work on police bias in Germany, in particular experimental research with active‐duty officers, remains scarce (Hunold & Singelnstein, 2022). In addition to documenting bias, studying police bias in Germany provides a crucial test of whether well‐established psychological mechanisms such as shooter bias and threat stereotypes extend beyond U.S.‐centric contexts. It allows researchers to examine their generalizability across Western policing cultures, their variation in relation to Germany's specific historical and demographic dynamics, and how racialized categories such as ‘Arab’ or ‘Muslim’ may shape bias differently in Europe than categories such as ‘Black’ in the United States. The present study addresses these gaps by providing multi‐method data on German police officer bias.

We adopted a multifaceted approach to operationalize bias, focusing on negative prejudice, stereotypes and discriminatory behavioural tendencies. Prejudice was assessed through self‐reported preferences for Arab versus White individuals (Axt, 2017). We hypothesized that officers would report stronger preference for White than for Arab individuals. Stereotypes were measured by ratings of the perceived trustworthiness of individuals perceived as Arab versus White. Drawing on prior research indicating threat‐related stereotypes towards Arab individuals (Stelter et al., 2023), we hypothesized that police officers would judge Arab individuals as less trustworthy than White individuals. Discriminatory behavioural tendencies were assessed using an adapted first‐person shooter task (Correll et al., 2002; Essien et al., 2017). Here, we hypothesized faster reactions to armed Arab versus White targets and slower reactions to unarmed Arab versus White targets, along with a lower threshold for detecting weapons on Arab targets. In addition to examining average sample levels of intergroup bias, we assessed distributions of bias to explore whether bias is prevalent across the sample or whether it is confined to a small group of individuals.

In addition to assessing bias, the present research explores psychological factors that may explain police officer bias. We measured social dominance orientation and internal motivation to suppress prejudice as indicators of inter‐individual differences in intergroup ideologies and motivations. Furthermore, we assessed external motivation to suppress prejudice and perceived norms regarding racist comments as measures of institutional norms. Lastly, we also assessed construal of intergroup contact with individuals perceived as Arab as an indicator of specific on‐the‐job experiences. By examining relationships between police officer bias and intergroup ideologies, individual motivations, institutional culture and intergroup contact, we aimed at disentangling factors linked to police officer bias.

To determine if the observed effects were specific to police officers, we also recruited civilian participants. We hypothesized that, similar to police participants, civilian participants would prefer White over Arab individuals, judge Arab individuals as less trustworthy, and show a shooter‐bias pattern with faster responses to armed Arab than White targets, slower responses to unarmed Arab than White targets, and a lower threshold for indicating the presence of weapons on Arab targets. We explored whether these biases towards racialized groups and related factors—intergroup ideologies, motivations, perceived norms and intergroup contact—differed between police officers and civilians. Additionally, we explored if potential differences in prejudice, stereotypes and behaviours between police officers and civilians were mediated by these factors.

## METHODS

### Transparency and openness

Data collection procedures, hypotheses and analyses were preregistered via the Open Science Framework (OSF). The study was approved by the local ethics committee of the FernUniversität in Hagen. Data and analysis code are available on OSF (https://osf.io/ndqwp). We report how we determined our sample size, all data exclusions (if any), all manipulations and all measures in the study.

### Data collection procedure

Police participants were recruited from July to August 2023 via police networks in southern Germany. A convenience sample of civilians was recruited from the student participant pool at FernUniversität in Hagen from August to September 2024. Participants were informed about the study and provided initial consent. They first completed the first‐person shooter task, followed by self‐report measures in a fixed order: trustworthiness ratings, social dominance orientation, preference measure, contact frequency and quality with individuals perceived as Arab, internal and external motivation to suppress prejudice, perception of norms regarding racist jokes and comments, and demographic information. After the study, participants were debriefed and gave consent for data analysis.

### Participants

We recruited *n* = 208 police officers and *n* = 237 civilian participants (total *N* = 445). We aimed for minimum sample sizes of *n* = 200 participants per group to obtain test power of 1‐β = 0.80 to detect a small effect size (dz = 0.18) in one‐sample and paired *t*‐tests, assuming α = .05 (one‐sided).

Demographic information of both participant groups is reported in Table 1.^2^ Civilian participants reported work experience in diverse professions (civil service *n* = 15; consulting and finance *n* = 17, engineering and IT *n* = 24; hospitality and tourism *n* = 7; marketing and sales *n* = 11, science and academia *n* = 10; social service *n* = 36, miscellaneous *n* = 80; student only *n* = 22). Two participants from the civilian sample reported to be police officers and were reassigned to the police sample.^3^

**TABLE 1:** Demographic information of police and civilian participants.

	Police(*n* = 208)	Civilian(*n* = 237)
Gender		
Female	52	141
Male	141	88
Other or not reported	15	8
Ethnicity		
Arab or Arab German	0	5
Asian or Asian German	1	5
Black or Afro‐German	0	2
Turkish or Turkish German	5	7
White	180	202
Other or not reported	22	16
Birth years		
1950–1969	35	29
1970–1979	57	32
1980–1989	57	68
1990–2009	58	108

### Material

#### First‐person shooter task

The first‐person shooter task, adapted from Correll et al. (2002), was integrated into an online survey using the MIND.set tool from the German Center for Integration and Migration Research (DeZIM). Participants decided if targets were holding a weapon or a harmless object (cup/phone) by pressing the A or L keys. The task included 8 practice trials and 80 main trials, using full body images of male targets (White and Arab) from the DeZIM Picture Database (Veit & Essien, 2022). Targets were presented in 20 different street scenes, with each trial starting with 1–3 backgrounds shown for 500–1000 milliseconds, followed by a target holding an object. Participants had 850 milliseconds to respond and received visual feedback after each trial. Scores were based on a pay‐off matrix (see Correll et al., 2002).

#### Trustworthiness rating

We used an exemplar‐based measure of perceived trustworthiness, where participants rated 14 male portraits (7 prototypically White, 7 prototypically Arab) on a scale from 1 (not at all trustworthy) to 7 (extremely trustworthy). Portraits were from the DEZIM Picture Database based on prototypicality ratings (Veit & Essien, 2022). Internal consistencies were satisfactory for police (White: Ω = 0.78, Arab: Ω = 0.80) and good for civilian participants (White: Ω = 0.83, Arab: Ω = 0.89).

#### Social dominance orientation

Social dominance orientation was measured using 16 items from the SDO6 scale (e.g. ‘No group should dominate in society’; Pratto et al., 1994), which were rated on a scale from 1 (strongly agree) to 7 (strongly disagree). We observed good internal consistencies are for police (Ω = 0.86) and civilian participants (Ω = 0.93).

#### Group preference

Prejudice towards Arab individuals was assessed using a one‐item preference measure (Axt, 2017), ranging from 1 (‘I have a strong preference for people perceived as Arab over White people’) to 7 (‘I have a strong preference for White people over those perceived as Arab’), with 4 indicating no preference.

#### Intergroup contact

The frequency of contact with individuals perceived as Arab was assessed by asking participants to estimate the percentage of such encounters in their professional and private lives over the past 6 months (0% to 100%). The quality of contact was measured by asking how often they had positive or negative experiences with Arab individuals, rated on a scale from 1 (never) to 7 (very often).

#### Perceived institutional norms

Perceived institutional norms regarding racist jokes and comments at work (and in private life for civilians) were measured using four statements rated from 1 (strongly agree) to 7 (strongly disagree). Examples include ‘Negative comments about ethnic and religious minorities are accepted at work’ and ‘My colleagues make racist jokes’ (see Váradi et al., 2020; Visintin et al., 2020). Internal consistencies were high: workplace norms among police participants (Ω = 0.90), workplace norms among civilian participants (Ω = 0.95) and private life norms among civilian participants (Ω = 0.90).

#### Internal and external motivation to suppress prejudice

We assessed internal and external motivation to suppress prejudice using a 10‐item questionnaire (Banse & Gawronski, 2003; Plant & Devine, 1998), with responses on a scale from 1 (strongly agree) to 7 (strongly disagree). Internal motivation included items like ‘I try to behave unprejudiced towards ethnic and religious minorities because it is personally important to me’, while external motivation included items like ‘I try to hide any negative thoughts about ethnic and religious minorities to avoid negative reactions from others’. Internal consistencies were satisfactory for police participants (internal: Ω = 0.79, external: Ω = 0.69) and good for civilian participants (internal: Ω = 0.86, external: Ω = 0.76).

##### Demographic questions

Participants reported their gender, decade of birth and identification with any of the following groups: ‘Arab or Arab German’, ‘Asian or Asian German’, ‘Black or Afro‐German’, ‘Turkish or Turkish‐German’, ‘White’ or ‘another category’. Police participants also provided job‐related information, such as the start of their service, career path, position, work schedule, current tasks, job‐specific training and work environment (see Supplement Tables S1–S4 for details).

## RESULTS

### Assessing bias: Group preference, trustworthiness ratings and shooter biases

In a set of preregistered analyses, we analysed police participants' preference for people perceived as White versus Arab, trustworthiness ratings and shooter biases in the first‐person shooter task. We also compared these biases between police and civilian participants.

#### Group preference

Both participant groups displayed a preference for White over Arab people, as indicated by responses above the scale midpoint of 4 for both police, M=4.64, 95% CI $\left[4.56,\infty \right]$, $t\left(204\right)=12.39$, p<.001 (preregistered as one‐sided test), dz = 0.87, 95% CI [0.70; 1.03] and civilian participants, M=4.63, 95% CI $\left[4.51,\infty \right]$, $t\left(235\right)=8.72$, p<.001 (preregistered as one‐sided test), dz = 0.57, 95% CI [0.43; 0.70]. There was no significant difference between police and civilian participants, $t\left(439\right)=-0.14$, p=.891, *d* = −0.01, 95% CI [−0.20; 0.17].

#### Trustworthiness rating

**FIGURE 1:**
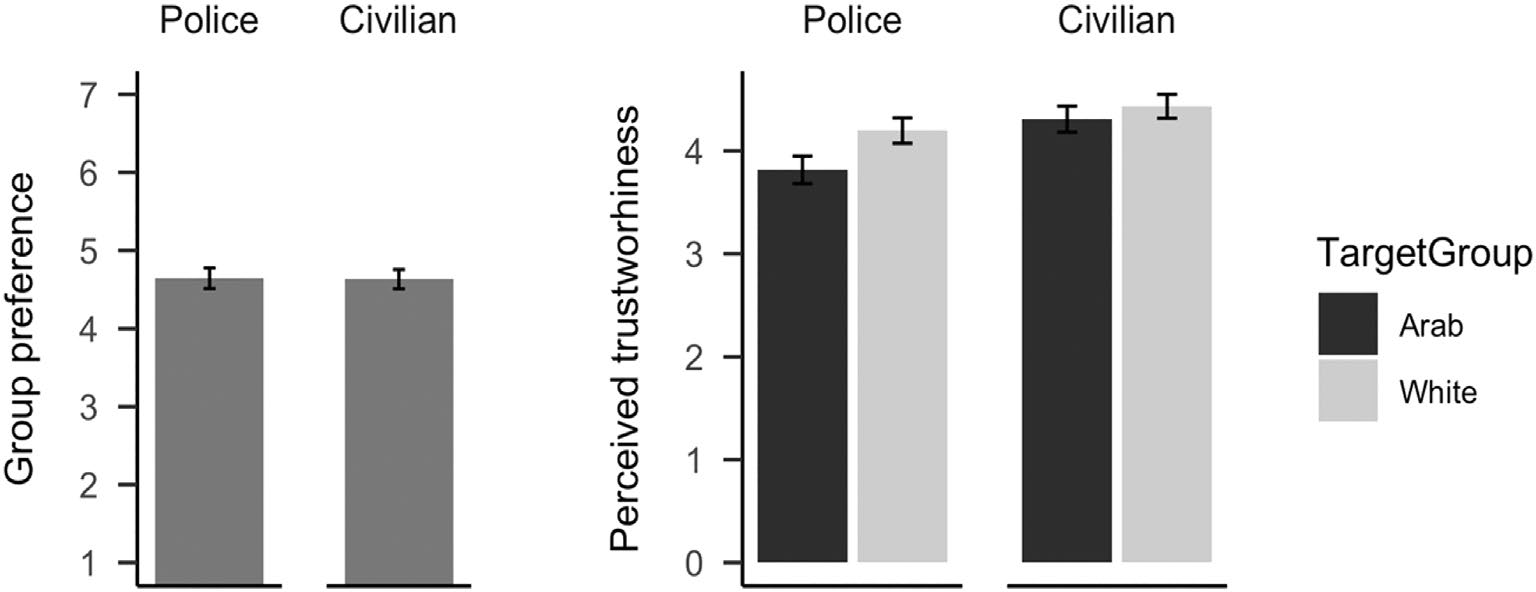
Police and civilian participants' preference of White vs. Arab individuals are displayed in the left panel. Preference scores below 4 indicate a preference for Arab individuals; scores above 4 in indicate a preference for White individuals. Police and civilian participants' trustworthiness ratings of Arab versus White individuals are displayed in the right panel.

Police participants rated Arab individuals as less trustworthy than White individuals, $t\left(200\right)=6.62$, p<.001 (preregistered as one‐sided), dz = 0.47, 95% CI [0.32; ∞]. Furthermore, police participants' trustworthiness ratings for Arab individuals were lower than the scale midpoint, M=3.81, 95% CI $\left[3.70,3.92\right]$, $t\left(201\right)=-3.33$, p<.001 (two‐sided), dz = −0.23, 95% CI [−0.37; −0.09]; for White individuals, trustworthiness ratings were higher than the scale midpoint, M=4.20, 95% CI $\left[4.09,4.32\right]$, $t\left(203\right)=3.61$, p<.001 (two‐sided), dz = 0.25, 95% CI [0.11; 0.39]. This indicates that, on average, police participants perceived White individuals as trustworthy, whereas they perceived Arab individuals as untrustworthy (see Figure 1).

Civilian participants perceived both Arab, M=4.32, 95% CI $\left[4.18,4.47\right]$, $t\left(228\right)=4.37$, p<.001 (two‐sided), dz = 0.29, 95% CI [0.16, 0.42], and White individuals, M=4.42, 95% CI $\left[4.30,4.55\right]$, $t\left(230\right)=6.69$, p<.001 (two‐sided), dz = 0.44, 95% CI [0.30; 0.57], as trustworthy, as indicated by tests against the scale midpoint. But they perceived Arab individuals as slightly less trustworthy than White individuals, $t\left(227\right)=1.96$, p=.025 (preregistered as one‐sided test), dz = 0.13, 95% CI [0.00; ∞]. Yet this difference in perceived trustworthiness was smaller for civilian than police participants, as indicated by a two‐way interaction in a mixed 2 (Participant Group; between participants) by 2 (Target Group; within‐participants) ANOVA. Also, civilian participants perceived all individuals as overall more trustworthy than police participants, $F\left(1,427\right)=21.17$, p<.001, ${\hat{\eta }}_{p}^{2}=.05$, 90% CI [.02, .08].

#### First‐person shooter task

We classified responses in the first‐person shooter task into hit and false alarm rates. Based on signal detection theory (Green & Swets, 1966), we calculated response bias c (−0.5 × (zHits + zFalse Alarms)), indicating participants' tendency to indicate the presence of a weapon. Hit rates = 1 or false alarm rates = 0 were adjusted following the procedure by Macmillan and Creelman (2005). Reaction times (RT) were averaged across participants and conditions for all analyses. For correlational analyses, we computed difference scores of RTs and c, as indices of the shooter bias according to the following formulas: (RT no gun/Arab target − RT no gun/White target) + (RT gun/Arab target − RT gun/White target), c White target − c Arab target.

**FIGURE 2:**
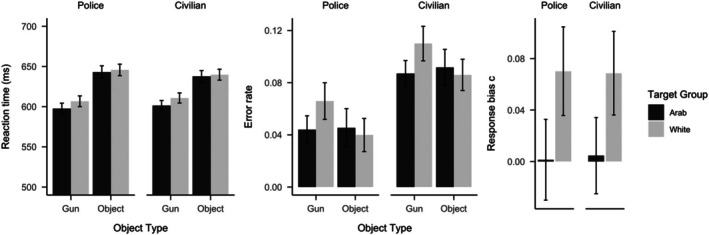
Shooter biases in reaction times, errors and response bias c (i.e. tendency to indicate the presence of a weapon). Error bars represent 95% confidence intervals.

We compared participants reaction times and errors to armed and unarmed Arab and White targets in two mixed 2 (Participant Group: police vs. civilian) by 2 (Target group: Arab vs. White) by 2 (Object Type: gun vs. no gun) ANOVAs with repeated measures on the last two factors (see Table 2 and Figure 2). Results showed significant Target Group × Object Type interactions for reaction times and errors, indicating shooter biases in both measures. This interaction was driven by fewer errors, $t\left(441\right)=-4.93$, p<.001, dz = −0.23, 95% CI [−0.33; −0.14], and faster responses, $t\left(440\right)=-7.17$, p<.001, dz = −0.34, 95% CI [−0.44; −0.24], to armed Arab compared to armed White targets. There were no significant differences in errors, $t\left(441\right)=1.36$, p=.088, dz = 0.06, 95% CI [−0.03; 0.16], or response times, $t\left(440\right)=-1.40$, p=.918, dz = −0.34, 95% CI [−0.16; 0.03], to unarmed Arab versus unarmed White targets. These findings indicate shooter biases in errors and response times for both, police and civilian participants.

**TABLE 2:** ANOVA results for reaction times and errors in the first‐person shooter task.

Effect	Reaction times						Errors					
	*η*_*p*_^2^	90% CI	*F*	df_1_	df_2_	*p*	*η*_*p*_^2^	90% CI	*F*	df_1_	df_2_	*p*
PG	.00	[.00, .01]	0.04	1	438	.833	.08	[.05, .13]	40.61	1	440	<.001
OT	.43	[.38, .48]	334.48	1	438	<.001	.02	[.00, .04]	7.96	1	440	.005
TG	.08	[.04, .12]	36.66	1	438	<.001	.02	[.00, .05]	8.66	1	440	.003
PG × OT	.01	[.00, .04]	5.51	1	438	.019	.00	[.00, .01]	0.11	1	440	.739
PG × TG	.00	[.00, .00]	0.00	1	438	.999	.00	[.00, .00]	0.01	1	440	.939
OT × TG	.03	[.01, .06]	11.72	1	438	<.001	.05	[.02, .09]	18.09	1	440	<.001
PG × OT × TG	.00	[.00, .01]	0.05	1	438	.816	.00	[.00, .00]	0.01	1	440	.928

Abbreviations: OT, object type; PG, participant group; TG, target group.

We also compared the response bias c parameter for Arab versus White targets as an indicator of participants' tendency to indicate the presence of a weapon in a mixed 2 (Participant Group: police vs. civilian) by 2 (Target: Arab vs. White) ANOVA with repeated measures on the last factor. Results showed that response bias c was more positive for White targets compared to Arab targets, $F\left(1,440\right)=18.88$, p<.001, ${\hat{\eta }}_{p}^{2}=.04$, 90% CI [.02, .08]. There was no difference in response bias c between police and civilian samples, $F\left(1,440\right)=0.00$, p=.962, and no interaction between Sample and Target group, $F\left(1,440\right)=0.00$, p=.876. Further *t*‐tests showed that response bias c was larger than zero for White targets, $t\left(441\right)=5.78$, p<.001, dz = 0.27, 95% CI [0.18; 0.37], and response bias c was not different from zero for Arab targets, $t\left(441\right)=0.28$, p=.779, dz = 0.01, 95% CI [−0.08; 0.11]. These findings suggest that both police and civilian participants were less inclined to indicate the presence of a weapon for White targets, while showing no such tendency for Arab targets.

### Distribution of group preference, trustworthiness ratings, and shooter biases

**FIGURE 3:**
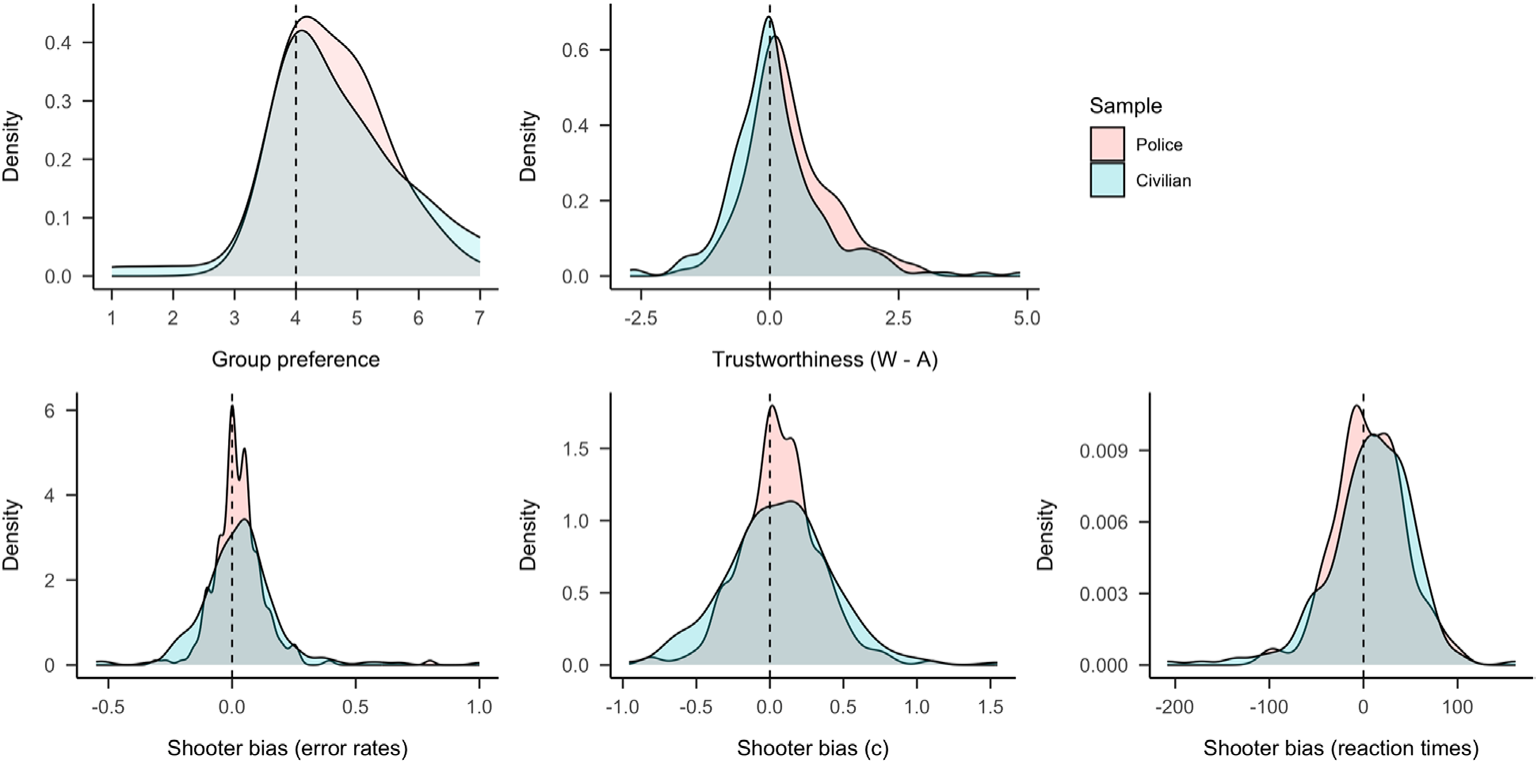
Density plots for prejudice, stereotypes and shooter biases separately for police and civilian participants. Note that values larger than the vertical dotted lines represent biases against Arab individuals.

We examined whether group preference, differences in perceived trustworthiness of White versus Arab individuals, and shooter biases in error rates, reaction times and response bias c (i.e. tendency to indicate the presence of a weapon) followed a normal distribution, or if distributions were skewed towards biased responses. Skewness and kurtoses of bias measures reported in Table 3. Density plots are depicted in Figure 3.

**TABLE 3:** Descriptive statistics and distribution indices of prejudice, stereotype and shooter bias measures.

	Min	Max	Mean	(SD)	Md	Skewness	Kurtosis
Police							
Group preference	3.00	7.00	4.64	0.74	5.00	0.82	0.12
Trustworthiness (W‐A)	−1.71	2.86	0.38	0.82	0.29	0.64	0.59
Shooter bias (error rates)	−0.31	0.80	0.03	0.11	0.00	1.87	12.82
Shooter bias (c)	−0.84	1.10	0.07	0.27	0.02	0.10	1.25
Shooter bias (reaction times)	−104.50	102.60	6.41	37.25	5.09	−0.08	0.31
Civilian							
Group preference	1.00	7.00	4.63	1.11	4.00	−0.07	1.21
Trustworthiness (W‐A)	−2.71	4.86	0.12	0.95	0.00	1.18	4.20
Shooter bias (error rates)	−0.55	1.00	0.03	0.16	0.03	1.16	7.20
Shooter bias (c)	−0.96	1.55	0.06	0.36	0.06	0.24	0.93
Shooter bias (reaction times)	−208.49	161.63	7.35	46.00	11.06	−0.94	2.97

Most measures were somewhat right skewed with a longer tail towards more biased responses; yet all values were within the range of acceptable skewness for normal data (−2 to +2; Hair et al., 2010). Also, all measures were somewhat peaked as indicated by positive kurtosis values; yet all values except for the shooter bias in error rates were still acceptable for normal data (−7 to +7, Hair et al., 2010). Taken together, these results indicate that all bias measures can be considered approximately normally distributed, suggesting that bias was not confined to a subset of participants.

### Predictors of biases

We explored relationships between social dominance orientation, motivation to suppress prejudice, perceived institutional norms and intergroup contact with group preference, trustworthiness ratings and shooter biases. For descriptive statistics and group comparisons of predictor variables, see Table 4. Police and civilian participants differed in levels of social dominance orientation and intergroup contact. Police participants had significantly higher levels of social dominance orientation and reported more on‐the‐job contact with individuals perceived as Arab, which was more negative and less positive. In their private lives, police participants had less overall and less positive contact, but similar levels of negative contact compared to civilians.

**TABLE 4:** Descriptive statistics and group comparisons of predictor variables.

Measure	Police		Civilian		*t*	df	*p*	dz	95% CI
	*M*	SD	*M*	SD					
SDO	2.59	0.83	2.32	1.00	3.11	443	.002	0.30	[0.11; 0.48]
IMS	5.75	0.95	5.70	1.18	0.56	443	.579	0.05	[−0.13; 0.24]
EMS	3.82	1.32	3.61	1.31	1.69	443	.092	0.16	[−0.03; 0.35]
Norms (job)	5.22	1.40	5.32	1.61	−0.72	443	.470	−0.07	[−0.26; 0.12]
Norms (private)	6.12	0.88	4.79	1.48	—	—	—	—	—
Contact freq. (job)	31.54	25.28	21.79	21.77	4.37	443	<.001	0.42	[0.23; 0.60]
Pos. contact (job)	3.54	1.46	4.03	2.04	−2.86	437	.004	−0.27	[−0.46; −0.09]
Neg. contact (job)	4.15	1.80	2.31	1.58	11.43	436	<.001	1.09	[0.89; 1.30]
Contact freq. (private)	14.53	13.64	24.54	21.31	−5.81	443	<.001	−0.55	[−0.74; −0.36]
Pos. contact (private)	4.05	1.90	4.43	1.84	−2.09	440	.037	−0.20	[−0.39; −0.01]
Neg. contact (private)	2.72	1.47	2.54	1.60	1.17	440	.241	0.11	[−0.08; 0.30]

Abbreviations: EMS, external motivation to suppress prejudice; IMS, internal motivation to suppress prejudice; SDO, social dominance orientation.

Intercorrelations between all measures can be seen in the Supplement (Table S5). Because shooter bias indices were largely uncorrelated with any of the predictor variables,^4^ the following analyses focus on prejudice and stereotypes as outcome variables.

We conducted several exploratory multiple‐regression analyses. Outcome variables were preference for Whites and trustworthiness ratings of Arab individuals (controlling for trustworthiness ratings of individuals perceived as White). We sequentially included social dominance orientation, internal motivation to suppress prejudice, institutional culture (i.e. external motivation to suppress prejudice and perceived institutional norms), and intergroup contact frequency, and intergroup contact valence as predictors (see Tables 5 and 6).^5^

**TABLE 5:** Regression models for police participants.

	Preference for whites					Trustworthiness for Arab individuals				
	Model 1	Model 2	Model 3	Model 4	Model 5	Model 1	Model 2	Model 3	Model 4	Model 5
(Intercept)	4.081***	6.086***	6.241***	6.252***	6.552***	2.479***	0.895	1.022	1.047	1.318*
SDO	0.218***	0.047	0.051	0.05	0.039	−0.218***	−0.078	−0.073	−0.048	−0.027
IMS		−0.271***	−0.247***	−0.237***	−0.23***		0.216***	0.224***	0.22***	0.212**
EMS			−0.015	−0.017	−0.02			−0.026	−0.01	−0.016
Norms Job			−0.047	−0.053	−0.052			−0.012	−0.01	−0.015
Contact freq. (job)				0.001					−0.005*	
Contact freq. (private)				−0.004					0.001	
Pos. contact (job)					−0.096*					0.078
Neg. contact (job)					−0.007					−0.072*
Pos. contact (private)					0.005					−0.034
Neg. contact (private)					0.01					−0.013
Trust White							0.448***	0.443***	0.441***	0.397***

*Note*: Models predicting trustworthiness for Arab individuals control for Trustworthiness for White individuals.

Abbreviations: EMS, external motivation to suppress prejudice; IMS, internal motivation to suppress prejudice; SDO, social dominance orientation; Trust White, Trustworthiness for White individuals.

*p < .05; **p < .01; ***p < .001.

**TABLE 6:** Regression models for civilian participants.

	Preference for whites					Trustworthiness for Arab individuals				
	Model 1	Model 2	Model 3	Model 4	Model 5	Model 1	Model 2	Model 3	Model 4	Model 5
(Intercept)	4.052***	5.544***	5.057***	5.51***	5.127***	2.276***	0.347	−0.179	−0.8	−0.336
SDO	0.249***	0.081	0.031	0.02	−0.009	−0.31***	−0.088	−0.06	−0.035	−0.034
IMS		−0.193*	−0.226*	−0.251**	−0.146		0.255***	0.223**	0.252***	0.174*
EMS			0.181***	0.173**	0.187***			0.004	0.016	−0.009
Norms Job			0.026	0.022	0.015			0.129***	0.133***	0.159***
Contact freq. (job)				−0.001					0.006*	
Contact freq. (private)				−0.008*					0.005	
Pos. contact (job)					−0.037					−0.048
Neg. contact (job)					−0.102					0.081
Pos. contact (private)					−0.097*					0.134***
Neg. contact (private)					0.161**					−0.088
Trust White							0.612***	0.599***	0.621***	0.575***

*Note*: Models predicting trustworthiness for Arab individuals control for Trustworthiness for White individuals.

Abbreviations: EMS, external motivation to suppress prejudice; IMS, internal motivation to suppress prejudice; SDO, social dominance orientation; Trust White, Trustworthiness for White individuals.

*p < .05; **p < .01; ***p < .001.

#### Intergroup ideology: Social dominance orientation

We included social dominance orientation as the first predictor (see Model 1, Tables 5 and 6). Results showed that social dominance orientation predicted preference for Whites and trustworthiness of Arab individuals. This pattern of results was the same for police and civilian participants.

#### Motivation: Internal motivation to suppress prejudice

We included internal motivation to suppress prejudice as another predictor (see Model 2, Tables 5 and 6). Results showed that motivation to suppress prejudice as another predictor predicted preference for Whites and trustworthiness of Arab individuals. Social dominance orientation was no longer significant. This pattern of results was the same for police and civilian participants.

#### Institutional culture: External motivation to suppress prejudice and perceived institutional norms

We added external motivation to suppress prejudice and perceived institutional norms as further predictors (see Model 3, Tables 4 and 5). Results showed that only for civilian participants, external motivation to suppress prejudice predicted preference for Whites, and perceived institutional norms predicted differences in reported trustworthiness of Arab versus White individuals. For both police and civilian participants, internal motivation to suppress prejudice remained a significant predictor of preference for Whites and differences in reported trustworthiness of Arab versus White individuals.

#### Construal of intergroup contact

We added contact frequency as well as positive and negative contact on the job and in private life as further predictors (see Models 4 and 5, Tables 4 and 5). Relationships between contact measures and prejudice and stereotypes differed for police and civilian participants. For police participants, contact on‐the‐job was the most important predictor: More positive on‐the‐job contact predicted less preference for Whites and more negative on‐the‐job contact predicted lower levels of perceived trustworthiness for Arab individuals. Among civilian participants, positive and negative contact in their private lives predicted both their preference for Whites and their trustworthiness ratings of Arab individuals.

#### Social dominance orientation and negative contact during the job as mediators for perceived trustworthiness of Arab individuals

**FIGURE 4:**
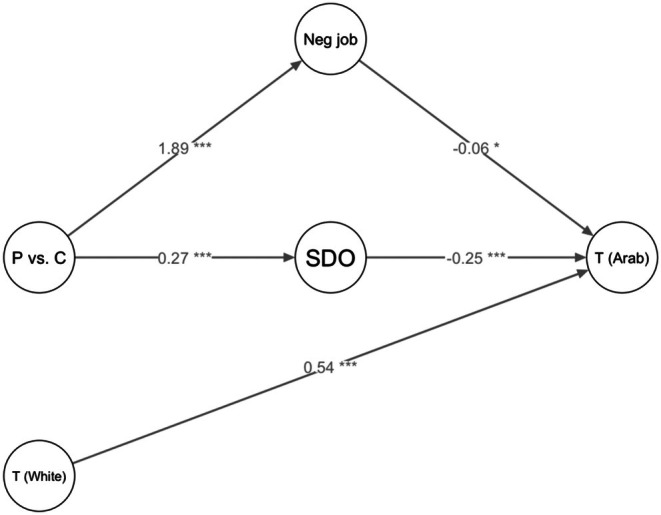
Social dominance orientation and negative contact on‐the‐job mediate differences between police versus civilian participants in trustworthiness ratings of Arab individuals. The model controlled for trustworthiness ratings of individuals perceived as White. P vs. C = police vs. civilian participants are included as dummy‐coded variable; SDO = social dominance orientation; Neg job = negative contact on‐the‐job; T (Arab) = trustworthiness of Arab individuals; T (White) = trustworthiness of individuals perceived as White.

In an exploratory mediation model, we examined whether police participant's higher levels of social dominance orientation and more negative on‐the‐job contact mediated differences between police and civilian participants in perceived trustworthiness of Arab individuals (see Figure 4). We included the categorical variable ‘participant group’ (police vs. civilian) as dummy‐coded variable in the model. The analysis was performed using the lavaan package in R with bootstrapping (1000 samples) to estimate the confidence intervals for the indirect effects.

The direct effect of participant group on trustworthiness ratings of Arab individuals was significant, *b* = −0.18, 95% CI [−0.34; −0.01], *p* = .028. The direct effect of social dominance orientation on trustworthiness ratings of Arab individuals was also significant, *b* = −0.25, 95% CI [−0.35; −0.13], *p* = <.001, as was the effect of negative on‐the‐job contact on trustworthiness ratings of Arab individuals, *b* = −0.06, 95% CI [−0.11; −0.01], *p* = .018. Trustworthiness of individuals perceived as White was a significant control variable, *b* = 0.54, 95% CI [0.42; 0.65], *p* = <.001. The total effect of participant group on trustworthiness ratings of Arab individuals was also significant, *b* = −0.06, 95% CI [−0.11; −0.01], *p* = .018.

Importantly, the indirect effect of participant group on trustworthiness ratings of Arab individuals through social dominance orientation was significant, *b* = −0.07, 95% CI [−0.12; −0.02], *p* = .005. The indirect effect through negative contact at work was also significant, *b* = −0.11, 95% CI [−0.22; −0.02], *p* = .023. This suggests that both social dominance orientation and negative on‐the‐job contact mediated differences in trustworthiness ratings of Arab individuals between police versus civilian participants.

## DISCUSSION

The present research investigated police bias and its underlying psychological processes using a multi‐method approach. We assessed multiple forms of bias, including group preference, perceptions of trustworthiness and behavioural tendencies in a first‐person shooter task. Our findings revealed biases among both police officers and civilians towards Arab individuals across various measures, including self‐reports and indirect behavioural assessments. In line with our hypotheses, police officers reported a preference for White people over those perceived as Arab (dz = 0.87) and rated Arab individuals as untrustworthy compared to White individuals (dz = 0.48). Additionally, in a first‐person shooter task, police officers responded faster and made fewer errors when reacting to armed Arab versus armed White targets and were more likely to indicate the presence of a weapon for Arab than for White targets. Taken together, these results suggest that police officers display biases towards Arab individuals.

Exploratory group comparisons indicated that civilian participants displayed similar levels of prejudice and discriminatory behavioural tendencies as police officers. There were no significant differences between these samples in terms of prejudice or behavioural tendencies in the first‐person shooter task. The only notable difference emerged in trust‐related judgements: police officers rated Arab individuals as untrustworthy than White individuals, and this difference in perceived trustworthiness between Arab and White individuals was slightly more pronounced than in the civilian sample. Although modest in size, this pattern suggests that trust‐related perceptions may play a distinct role in officers' evaluations of racialized individuals. Such dynamics are consistent with research showing that police officers sometimes interpret racialized faces as more suspicious or criminal‐looking (Eberhardt et al., 2004) and that distrust can shape how interactions unfold or whom police officers choose to monitor (Swencionis & Goff, 2017). Future work should examine the implications of these trust‐related perceptions, including how they contribute to racial profiling or differential treatment in everyday police work.

The findings of this study also contribute to the ongoing debate about the prevalence of bias among police officers. Previous research has questioned whether discriminatory behaviour is widespread among police officers or confined to a few individuals (Chalfin & Kaplan, 2021). We observed that bias measures were within acceptable ranges for normal data distribution (Hair et al., 2010). This suggests that bias was not limited to a few individuals. These results align with recent analyses indicating that discriminatory behaviour in judicial decisions and the job and housing markets is prevalent across the population (Galvan et al., 2026). Therefore, addressing police bias may require a systemic approach rather than focusing solely on few individual officers.

While the sample of police officers exhibited biases on average, there were also individual differences, suggesting that individual psychological characteristics still play a significant role in shaping biases. Our findings indicate that police officers with higher levels of social dominance orientation and lower levels of internal motivation to suppress prejudice were more distrustful of Arab individuals and reported more prejudice. Police officers reported higher social dominance orientation than civilians, but both groups had similar internal motivation to suppress prejudice. Additionally, the relationships between social dominance orientation, internal motivation and prejudice and stereotypes were similar for both police officers and civilians. Regression models showed that internal motivation to suppress prejudice was a stronger predictor of group preference than social dominance orientation. This suggests that motivations may be more relevant in shaping police officers' bias than intergroup ideologies. External motivation to suppress prejudice and perceived norms about racist comments were less important predictors of police officers' prejudice and stereotypes. This pattern provides limited support for the role of institutional or cultural norms (e.g. ‘cop culture’) in shaping individual biases in this context. It also opens up the possibility that approaches targeting internalized motivations may be more effective in mitigating bias than compliance‐based approaches.

Another important predictor of police officers' biases was their construal of intergroup contact. Negative construal of job‐related contact with individuals perceived as Arab was related to more distrust of Arab individuals and positive construal of job‐related contact with Arab individuals were linked to lower levels of prejudice. In contrast, intergroup contact in private contexts was unrelated to police officers' prejudice and stereotypes, suggesting that job‐related contact may dominate or even overwrite positive contact experiences in police officer's private lives. In line with this interpretation, differences between police versus civilians' (dis)trust for Arab individuals were mediated by construal of negative contact on‐the‐job. Due to the cross‐sectional nature of the study design, there are several ways to interpret relationships between police officers' negative construal of intergroup contact and bias.

One possible explanation for police officers' biases is that, due to the nature of their work, they may more frequently encounter negative contact situations with individuals from racialized groups (e.g. Fassin, 2013). This would suggest that negative intergroup contact may causally contribute to the development of prejudice and stereotypes. However, an alternative explanation is that pre‐existing biases among police officers may shape how they perceive these encounters. In other words, officers who are more prejudiced may be more likely to interpret interactions with Arab individuals as negative, regardless of the objective quality of the encounter. This interpretation aligns with findings that the initial words spoken by police officers to Black drivers during traffic stops can predict whether the interaction escalates (Rho et al., 2023; Voigt et al., 2017). Moreover, even when the objective quality of interactions is similar across racialized and non‐racialized groups, police officers' biases may still influence their construal of these encounters—leading them to perceive them as more negative. This highlights the complex, bidirectional relationship between bias and intergroup contact, where not only may contact shape attitudes, but attitudes may also shape the perception of contact (Binder et al., 2009).

The present research investigated police bias by focusing on individual officers, based on the rationale that individual biases may shape police officer's perceptions and behaviour, which in turn may shape community perceptions of the police. However, focusing solely on individual biases has limitations. It may overlook systemic factors, such as departmental policies, training protocols or performance incentives, that guide officers' decisions. For instance, stop‐and‐frisk policies or quotas for arrests can institutionalize biased practices, regardless of an individual officer's intentions. Consistent with this critique, some scholars argue that addressing bias requires a broader lens, one that includes institutional frameworks (e.g. Camp, 2024). For example, changing how police departments evaluate officer performance (e.g. rewarding de‐escalation rather than arrests) could reduce biased outcomes more effectively than trying to change individual attitudes alone. Similarly, Chater and Loewenstein (2023) suggest that system‐level reforms—such as revising use‐of‐force policies, implementing transparent accountability mechanisms or restructuring training to emphasize empathy and procedural justice—may be more impactful in reducing disparities in policing outcomes than interventions targeting individual bias.

Another important aspect to address is that the shooter task showed low internal reliability, limiting its use for correlational analyses. Although we observed consistent shooter biases among both samples, we were thus unable to identify potential predictors of these biases. The robustness of shooter bias in the absence of measurement reliability may appear contradictory at first sight. However, this ‘reliability paradox’ (Hedge et al., 2018, p. 1166) is well known, such that experimental tasks that produce robust and replicable group effects fail to capture reliable individual differences. At the root of this paradox are the same factors that make these tasks effective at showing consistent experimental effects: low variability between participants, which also leads to low reliability when measuring individual differences. One methodological factor that makes responses in the first‐person shooter task particularly difficult to compare between individuals is a task structure that randomizes trial order, number of backgrounds and background type between participants. Each target is thus presented in a different context for each participant. One approach to increase reliability in the first‐person shooter task may thus be the use of a less complex task structure with less procedural variation between participants. Lastly, it is also possible that administrating the shooter task in an online setting may introduce additional noise compared to more controlled lab settings.

Some further limitations are worth noting. First, the analyses of individual differences were confined to measures of prejudice and stereotypes. Potentially due to the very low internal consistencies of the first‐person shooter task (below r = .15, see also Payne & Correll, 2020), shooter biases showed little correlation with self‐report measures. Although we observed consistent shooter biases among both samples, we were thus unable to identify potential predictors of these biases. Second, although the sample of police officers recruited for this study was larger than those in other studies (e.g. Correll, Park, Judd, & Wittenbrink, Sadler, & Keese, 2007; Gatto et al., 2010; but see Wortley & Homel, 1995) and included police officers from different task forces, the sample was not representative of all police officers. Additionally, participation in the study was voluntary, potentially introducing self‐selection biases. To gain a comprehensive understanding of biases in policing, future research should recruit larger, demographically representative samples, systematically including members from various police subgroups.

Despite these limitations, by employing a multitude of different measures, the present research provides robust evidence that police officers, as a reflection of society, exhibit pervasive biases towards racialized groups, which may impact their interactions with minoritized communities.

## AUTHOR CONTRIBUTIONS

Marleen Stelter: Conceptualization, methodology, writing—original draft preparation, data analysis. Iniobong Essien: Conceptualization, methodology, writing—review and editing. Jan Malte von Bargen: Conceptualization, methodology, writing—review and editing. Oliver Christ: Conceptualization, methodology, writing—review and editing.

## CONFLICT OF INTEREST STATEMENT

One co‐author is employed in a senior administrative role within a German state interior administration and assisted with participant recruitment by distributing the study invitation to police officers. Participation was voluntary, and no institution or employer had any role in the study design, data analysis, interpretation of the findings, or publication decisions. The authors declare no competing interests.

## Supporting information


**Table S1.** MANOVAs for different police subgroups.
**Table S2.** Means and ANOVA Results for different police task subgroups.
**Table S3.** Means and ANOVA Results for different police shift subgroups.
**Table S4.** Means and ANOVA Results for different age groups within the police.
**Table S5.** Zero‐order correlations of contact measures with shooter biases, prejudice and stereotypes for police (lower triangle) and civilian (upper triangle) participants.
**Figure S1.** Preference for White (vs. Arab) individuals in police and civilian participants controlling for age and gender.
**Table S6.** Analyses controlling for age and gender. ANOVA results comparing police and civilian participants' preference for White (vs. Arab) individuals controlling for participant age group and gender.
**Figure S2.** Perceived trustworthiness for Arab and White individuals in police and civilian participants controlling for age and gender.
**Table S7.** ANOVA results comparing police and civilian participants' trustworthiness for Arab versus White individuals controlling for participant age group and gender.
**Table S8.** ANOVA results for reaction times and errors in the first‐person shooter task controlling for participant age group and gender.

## Data Availability

Data collection procedures, hypotheses and analyses were preregistered via the Open Science Framework (OSF; see copies in supplement). Data and analysis code are available on OSF (https://osf.io/ndqwp).

## Footnotes

^1^
It is important to note that in the present article, we use the term “Arab individuals” to refer to individuals who are perceived as Arab based on visual features. We do not use it as an essentializing term for a homogenous group. Also, we do not suggest that these individuals would describe themselves as Arab.

^2^
Note that while students from FernUniversität in Hagen tend to be older and more diverse in educational backgrounds than average German students (FernUniversität in Hagen, 2024), participants from the civilian sample were younger and more female than police participants. Based on reviewers' suggestions, we conducted additional exploratory analyses, in which we added age and gender as covariates (see Supplement S6–S8 and Figures S1 and S2). The overall pattern of results for police–civilian comparisons remained the same after controlling for age and gender.

^3^
This participant reassignment did not change the results in any meaningful ways.

^4^
We observed very low internal consistencies for shooter bias indices in reaction times (*r* = .01, 95% CI [−.15; .15]), errors (*r* = .10, 95% CI [−.15; .28]) and c (*r* = .19, 95% CI [.08; .31]), as estimated via bootstrapped split‐half reliabilities. For police participants, none of the predictors were correlated with shooter bias indices (*r*s < |.10|; *p*s > .11; see Table S5). For civilian participants, the shooter bias in error rates correlated with SDO (*r* = −.14) and EMS (*r* = −.19) and the shooter bias response bias c correlated with EMS (*r* = −.18) and with SDO (*r* = −.13 and *r* = −.15).

^5^
Multicollinearity checks indicated only moderate correlations between predictors. Across all models, variance inflation factors (VIF) were below 2.6, suggesting that observed effects are not affected by multicollinearity.
