# Supplementary material for: Police officers' prejudice and distrust towards racialized groups is related to internal motivation to suppress prejudice and negative intergroup contact
Source: Br J Soc Psychol. 2026 May 15;65:e70094. doi: 10.1111/bjso.70094 (PMC13179431; doi:10.1111/bjso.70094)
Supplement: Supplementary file 1 — Table S1. MANOVAs for different police subgroups. Table S2. Means and ANOVA Results for different police task subgroups. Table S3. Means and ANOVA Results for different police shift subgroups. Table S4. Means and ANOVA Results for different age groups within the police. Table S5. Zero‐order correlations of contact measures with shooter biases, prejudice and stereotypes for police (lower triangle) and civilian (upper triangle) participants. Figure S1. Preference for White (vs. Arab) individuals in police and civilian participants controlling for age and gender. Table S6. Analyses controlling for age and gender. ANOVA results comparing police and civilian participants' preference for White (vs. Arab) individuals controlling for participant age group and gender. Figure S2. Perceived trustworthiness for Arab and White individuals in police and civilian participants controlling for age and gender. Table S7. ANOVA results comparing police and civilian participants' trustworthiness for Arab versus White individuals controlling for participant age group and gender. Table S8. ANOVA results for reaction times and errors in the first‐person shooter task controlling for participant age group and gender. [file BJSO-65-0-s001.docx]

# Supplementary material

## Comparison of subgroups within the police

We asked police participants about the start of police service (before 1980, 80-89, 90-99, 2000-2009, 2010-2014, 2015-2019, 2020 or later), career path (middle service/higher service with bachelor’s degree/senior service with master’s degree), position (uniformed police/criminal police), work schedule model (shift work/rotating shift/day service), current activity (traditional police work, special police tasks, cross-sectional tasks), minimum times for job-specific (deployment) training (0-10, 10-19, 20-29, 30-39, 40 or more), and place of service/work environment (more urban/more rural).

Police participants reported on their career paths as being medium-level service (*n* = 43), higher service (*n* = 147), or senior service (*n* = 14). Most police described their position as uniformed police (*n* = 148) or criminal investigation police (*n* = 62). N = 126 reported to work on day service, *n* = 62 reported to be on a rotating shift (early, late, and night), and *n* = 16 reported their work schedule model to be regular shift work (6am - 11pm). They described their current activity as either traditional police work (*n* = 128), cross-sectional tasks (*n* = 72), or special police tasks (*n* = 4). N = 44 reported their work environment as more urban and *n* = 165 described their work environment as more rural.

We observed significant differences between measures for police participants working on different tasks (i.e., traditional, cross-sectional or special tasks). Follow-up analyses revealed that police participants who do traditional police work report more negative contact, $t\left( 140.19 \right)=8.07$, $p<.001$, *d* = 1.21, 95% CI [0.90; 1.52] during the job and in their privat life $t\left( 168.41 \right)=3.32$, $p=.001$, *d* = 0.47, 95% CI [0.18; 0.76] compared to the other groups, ; they also reported lower trustworthiness of individuals perceived as Arab compared to the other groups, $t\left( 174.87 \right)=-2.77$, $p=.006$, *d* = -0.39, 95% CI [-0.68; -0.10].

**Table S1**
*MANOVAs for different police subgroups*

| Police subgroup | Pillai | DF 1 | DF 2 | *F* | *p* |
| --- | --- | --- | --- | --- | --- |
| Position (uniformed or criminal investigation) | 0.125 | 16.000 | 170.000 | 1.517 | .098 |
| Career path (medium-, higher-, or senior- level) | 0.321 | 48.000 | 519.000 | 1.297 | .093 |
| Work environment (urban or rural) | 0.097 | 16.000 | 170.000 | 1.142 | .321 |
| Tasks (traditional, cross-sectional or special tasks) | 0.485 | 48.000 | 519.000 | 2.083 | < .001 |
| Shift model (day service, rotating shift, or regular shift) | 0.400 | 48.000 | 519.000 | 1.662 | .005 |
| Age group | 0.523 | 64.000 | 692.000 | 1.627 | .002 |

We also observed differences between measures for police participants with different shift models (i.e., day service, rotating shift, or regular shift). Participants who work in day service report overall less contact, $t\left( 152.92 \right)=-5.04$, $p<.001$, *d* = -0.74, 95% CI [-1.03; -0.45], and less negative contact, $t\left( 183.04 \right)=-5.56$, $p<.001$, *d* = -0.77, 95% CI [-1.07; -0.48], compared to the other groups. There were no differences in any of the other measures between the subgroups.

Lastly, we observed significant differences between measures for police participants of different age groups. There were linear trends indicating that younger participants reported more contact, $F\left( 1,201 \right)=21.94$, $p<.001$, $\hat{\eta}_{G}^{2}=.098$, 90% CI $\left[ .043,.168 \right]$, and more negative contact, $F\left( 1,198 \right)=13.59$, $p<.001$, $\hat{\eta}_{G}^{2}=.064$, 90% CI $\left[ .020,.127 \right]$, than older participants (see Table S4). Also, participants born between 1980 and 2009 reported lower trustworthiness of individuals perceived as Arab compared to the older age groups, $t\left( 84.29 \right)=-2.89$, $p=.005$, *d* = -0.69, 95% CI [-0.98; -0.39]. Finally, there was a linear trend indicating that older participants displayed larger reaction time biases in the first-person shooter task, $F\left( 1,201 \right)=4.77$, $p=.030$, $\hat{\eta}_{G}^{2}=.023$, 90% CI $\left[ .001,.068 \right]$.

**Table S2**
*Means and ANOVA Results for different police task subgroups*

| Measure | Cross-sectional tasks | Traditional task | Special tasks | df 1 | df 2 | *F* | *p* |
| --- | --- | --- | --- | --- | --- | --- | --- |
| Preference | 4.708 | 4.640 | 4.250 | 3 | 186 | 0.743 | .527 |
| Trustworthiness Arab | 3.976 | 3.688 | 4.321 | 3 | 186 | 2.767 | .043 |
| Trustworthiness White | 4.270 | 4.152 | 4.857 | 3 | 186 | 2.274 | .081 |
| Error bias | 0.038 | 0.023 | -0.021 | 3 | 186 | 1.059 | .368 |
| Reaction-time bias | 8.357 | 4.658 | 5.333 | 3 | 186 | 0.561 | .642 |
| c White - c Arab | 0.102 | 0.056 | -0.073 | 3 | 186 | 1.116 | .344 |
| SDO | 2.433 | 2.698 | 2.094 | 3 | 186 | 2.341 | .075 |
| IMS | 5.908 | 5.633 | 6.450 | 3 | 186 | 1.758 | .157 |
| EMS | 3.669 | 3.873 | 3.550 | 3 | 186 | 0.470 | .704 |
| Norms (job) | 5.191 | 5.242 | 4.562 | 3 | 186 | 0.868 | .459 |
| Contact freq. (job) | 18.069 | 38.398 | 38.750 | 3 | 186 | 10.315 | < .001 |
| Pos. contact (job) | 3.714 | 3.375 | 5.250 | 3 | 186 | 3.355 | .020 |
| Neg. contact (job) | 2.929 | 4.820 | 3.000 | 3 | 186 | 24.448 | < .001 |
| Contact freq. (private) | 12.347 | 15.945 | 10.000 | 3 | 186 | 1.344 | .261 |
| Pos. contact (private) | 4.028 | 4.008 | 5.000 | 3 | 186 | 0.436 | .727 |
| Neg. contact (private) | 2.306 | 2.936 | 1.500 | 3 | 186 | 3.583 | .015 |

*Note.* SDO = social dominance orientation; IMS = internal motivation to suppress prejudice; EMS = external motivation to suppress prejudice

**Table S3**
*Means and ANOVA Results for different police shift subgroups*

| Measure | Regular shifts | Day service | Rotating shifts | df 1 | df 2 | *F* | *p* |
| --- | --- | --- | --- | --- | --- | --- | --- |
| Preference | 4.867 | 4.728 | 4.459 | 3 | 186 | 2.208 | .089 |
| Trustworthiness Arab | 3.411 | 3.907 | 3.689 | 3 | 186 | 2.483 | .062 |
| Trustworthiness White | 4.029 | 4.196 | 4.247 | 3 | 186 | 0.231 | .875 |
| Error bias | 0.039 | 0.025 | 0.029 | 3 | 186 | 1.127 | .340 |
| Reaction-time bias | -2.864 | 10.584 | -0.283 | 3 | 186 | 1.080 | .359 |
| c White - c Arab | 0.122 | 0.051 | 0.086 | 3 | 186 | 1.620 | .186 |
| SDO | 2.703 | 2.477 | 2.805 | 3 | 186 | 2.361 | .073 |
| IMS | 5.500 | 5.840 | 5.626 | 3 | 186 | 0.819 | .485 |
| EMS | 4.238 | 3.708 | 3.848 | 3 | 186 | 0.794 | .499 |
| Norms (job) | 5.297 | 5.248 | 5.141 | 3 | 186 | 0.080 | .971 |
| Contact freq. (job) | 47.062 | 24.492 | 40.887 | 3 | 186 | 8.930 | < .001 |
| Pos. contact (job) | 3.750 | 3.581 | 3.355 | 3 | 186 | 1.089 | .355 |
| Neg. contact (job) | 4.688 | 3.629 | 5.000 | 3 | 186 | 11.546 | < .001 |
| Contact freq. (private) | 15.625 | 13.286 | 16.871 | 3 | 186 | 1.419 | .239 |
| Pos. contact (private) | 4.000 | 4.144 | 3.817 | 3 | 186 | 0.371 | .774 |
| Neg. contact (private) | 2.438 | 2.568 | 2.983 | 3 | 186 | 1.520 | .211 |

*Note.* SDO = social dominance orientation; IMS = internal motivation to suppress prejudice; EMS = external motivation to suppress prejudice

**Table S4**
*Means and ANOVA Results for different age groups within the police*

| Measure | 1950-1969 | 1970-1979 | 1980-1989 | 1990-2009 | df 1 | df 2 | *F* | *p* |
| --- | --- | --- | --- | --- | --- | --- | --- | --- |
| Preference | 4.515 | 4.714 | 4.673 | 4.655 | 4 | 185 | 0.903 | .463 |
| Trustworthiness Arab | 3.862 | 3.926 | 3.937 | 3.517 | 4 | 185 | 4.220 | .003 |
| Trustworthiness White | 4.143 | 4.268 | 4.265 | 4.126 | 4 | 185 | 0.586 | .673 |
| Error bias | 0.057 | 0.023 | 0.017 | 0.022 | 4 | 185 | 0.776 | .542 |
| Reaction-time bias | 17.434 | 6.962 | 5.758 | -1.016 | 4 | 185 | 2.971 | .021 |
| c White - c Arab | 0.146 | 0.047 | 0.050 | 0.056 | 4 | 185 | 0.528 | .715 |
| SDO | 2.633 | 2.711 | 2.481 | 2.593 | 4 | 185 | 0.533 | .712 |
| IMS | 5.939 | 5.733 | 5.747 | 5.628 | 4 | 185 | 0.963 | .429 |
| EMS | 3.455 | 3.905 | 3.779 | 3.924 | 4 | 185 | 0.597 | .665 |
| Norms (job) | 5.235 | 5.509 | 5.298 | 4.841 | 4 | 185 | 1.611 | .173 |
| Contact freq. (job) | 19.818 | 27.123 | 29.246 | 43.431 | 4 | 185 | 6.306 | < .001 |
| Pos. contact (job) | 3.667 | 3.375 | 3.702 | 3.456 | 4 | 185 | 0.823 | .512 |
| Neg. contact (job) | 3.182 | 4.196 | 3.965 | 4.786 | 4 | 185 | 4.722 | .001 |
| Contact freq. (private) | 10.030 | 14.702 | 15.684 | 15.776 | 4 | 185 | 0.949 | .437 |
| Pos. contact (private) | 4.424 | 3.544 | 4.509 | 3.873 | 4 | 185 | 2.543 | .041 |
| Neg. contact (private) | 2.455 | 2.643 | 2.772 | 2.768 | 4 | 185 | 0.479 | .751 |

*Note.* SDO = social dominance orientation; IMS = internal motivation to suppress prejudice; EMS = external motivation to suppress prejudice

**Table S5**
*Zero-order correlations of contact measures with shooter biases, prejudice and stereotypes for police (lower triangle) and civilian (upper triangle) participants*

|  | 1 | 2 | 3 | 4 | 5 | 6 | 7 | 8 | 9 | 10 | 11 | 12 | 13 | 14 | 15 |
| --- | --- | --- | --- | --- | --- | --- | --- | --- | --- | --- | --- | --- | --- | --- | --- |
| 1. Reaction-time bias | - | -.13 | -.12 | -.08 | -.12 | -.02 | -.01 | -.01 | -.01 | -.10 | -.03 | .00 | -.04 | .06 | .07 |
| 2. Error bias | -.05 | - | .94*** | -.07 | .15* | -.07 | -.03 | -.04 | -.07 | .01 | .00 | -.15* | .12 | -.18** | -.08 |
| 3. c White - c Arab | .00 | .95*** | - | -.05 | .10 | -.07 | -.02 | -.04 | -.06 | .01 | .00 | -.13* | .12 | -.17** | -.04 |
| 4. Preference | .00 | .01 | .00 | - | .32*** | -.11 | .05 | -.21** | -.15* | .21** | -.24*** | .23*** | -.26*** | .21*** | -.05 |
| 5. Trustworthiness | .00 | .11 | .08 | .31*** | - | -.20** | .07 | -.05 | -.16* | .20** | -.22*** | .29*** | -.35*** | -.01 | -.28*** |
| 6. Contact freq. (job) | -.11 | .05 | .07 | .06 | .18* | - | .40*** | .36*** | .48*** | .23*** | .17** | -.01 | -.07 | -.06 | -.04 |
| 7. Pos. contact (job) | -.09 | .06 | .07 | .14 | .20** | .64*** | - | .06 | .16* | .67*** | .03 | .28*** | -.34*** | .12 | -.26*** |
| 8. Neg. contact (job) | -.07 | .01 | .02 | -.28*** | -.17* | -.20** | -.40*** | - | .15* | -.01 | .55*** | -.21*** | .19** | -.06 | .07 |
| 9. Contact freq. (private) | .02 | .02 | .01 | -.08 | .00 | .29*** | .09 | .04 | - | .20** | .23*** | .07 | -.12 | -.05 | -.06 |
| 10. Pos. contact (private) | .10 | .04 | .02 | .12 | .12 | .13 | .25*** | -.20** | .15* | - | -.05 | .38*** | -.45*** | .06 | -.23*** |
| 11. Neg. contact (private) | .09 | .09 | .12 | -.18* | -.09 | -.03 | -.12 | .46*** | .15* | -.08 | - | -.22*** | .24*** | .01 | -.02 |
| 12. SDO | -.03 | .07 | .05 | .24*** | .14 | .22** | .27*** | -.25*** | .01 | .23** | -.21** | - | -.74*** | .12 | -.29*** |
| 13. IMS | .05 | -.03 | -.01 | -.37*** | -.23*** | -.13 | -.20** | .26*** | .08 | -.19** | .36*** | -.57*** | - | -.03 | .30*** |
| 14. EMS | -.05 | -.05 | -.05 | .02 | .05 | .19** | .18* | -.16* | .05 | .14* | -.11 | .13 | -.06 | - | -.03 |
| 15. Norms (job) | .03 | .07 | .08 | -.19** | -.13 | -.05 | -.02 | .09 | -.10 | -.05 | .09 | -.17* | .32*** | -.15* | - |

*Note.* * p < .05; ** p < .01; *** p < .001; SDO = social dominance orientation; IMS = internal motivation to suppress prejudice; EMS = external motivation to suppress prejudice

## Analyses controlling for age and gender

We ran additional exploratory analyses comparing bias measures for police and civilian participants while controlling for age and gender.

**Group preference.** Police and civilian participants showed similar levels of preference for White over Arab individuals after controlling for age and gender (Table S6 and Figure S1).

**Figure S1**
*Preference for White (vs. Arab) individuals in police and civilian participants controlling for age and gender.*


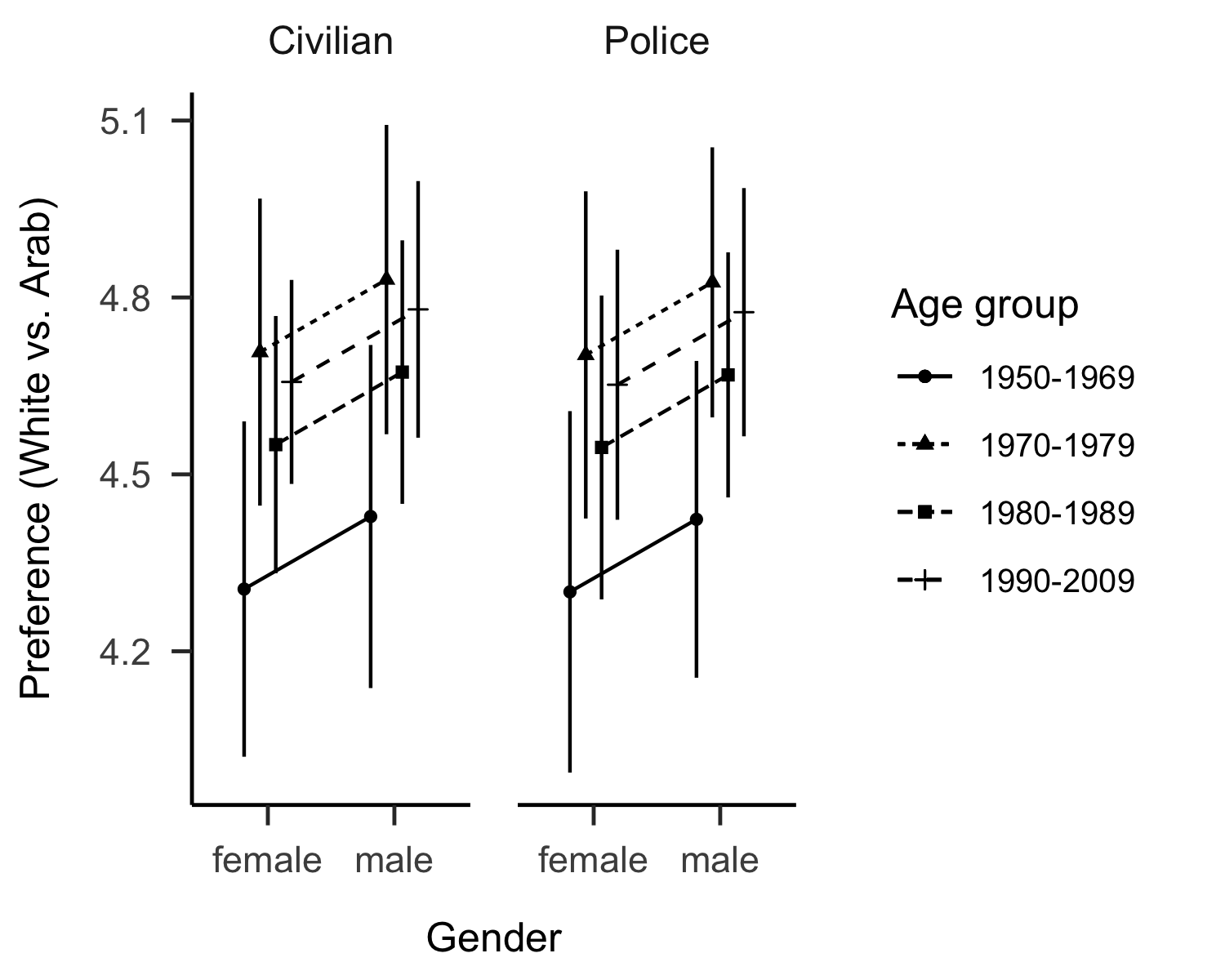


**Table S6**
*Analyses controlling for age and gender) ANOVA results comparing police and civilian participants’ preference for White (vs. Arab) individuals controlling for participant age group and gender*

| Effect | $\hat{\eta}_{p}^{2}$ | 90% CI | $F$ | ${df}_{1}$ | ${df}_{2}$ | $p$ |
| --- | --- | --- | --- | --- | --- | --- |
| Participant group | .00 | [.00, .00] | 0.00 | 1 | 411 | .963 |
| Gender | .00 | [.00, .02] | 1.43 | 1 | 411 | .233 |
| Age | .02 | [.00, .04] | 2.38 | 3 | 411 | .069 |

**Trustworthiness rating.** Results are reported in Table S7 and Figure S2. After controlling for age and gender, police participants reported overall lower levels of trustworthiness. The difference in perceived trustworthiness for Arab vs. White individuals was only marginally smaller for civilian than police participants, as the two-way interaction in a mixed 2 (Participant Group; between participants) by 2 (Target Group; within-participants) ANOVA was only marginally significant. Additionally, we observed an interaction effect between participant gender and target group, indicating that only male participants reported lower trust for Arab compared to White individuals (*M_D_* =0.43, 95% CI [0.31,0.54], *t*(218) = 7.10, *p* <.001), whereas female participants reported no difference in trust toward Arab and White individuals (*M_D_* = 0.04, 95% CI [-0.09,0.16], *t*(185) = 0.59, *p* = .555), and a main effect indicating that older participants reported higher levels of trustworthiness compared to younger participants.

**Figure S2**
*Perceived trustworthiness for Arab and White individuals in police and civilian participants controlling for age and gender.*


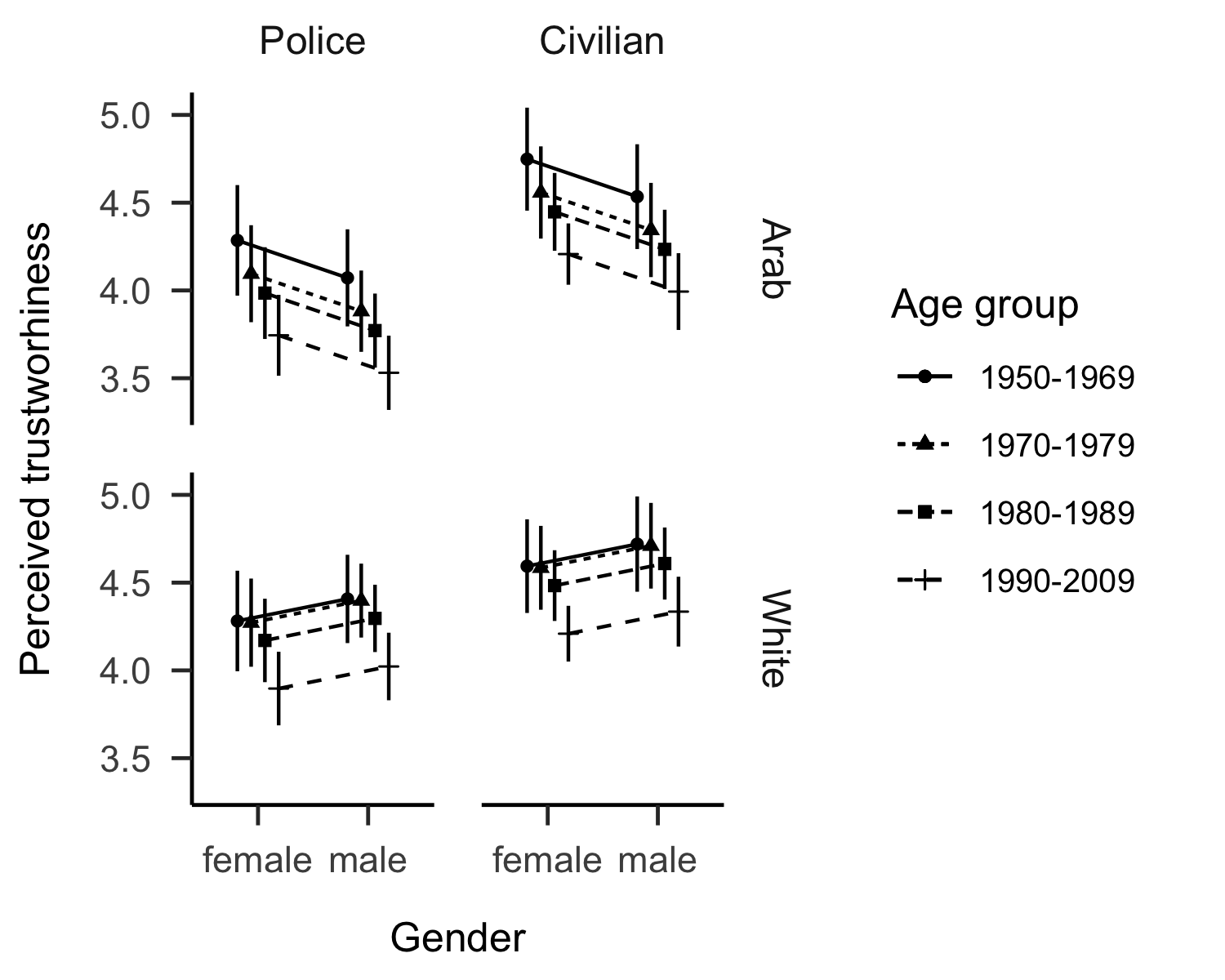


**Table S7**
*ANOVA results comparing police and civilian participants’ trustworthiness for Arab versus White individuals controlling for participant age group and gender.*

| Effect | $\hat{\eta}_{p}^{2}$ | 90% CI | $F$ | ${df}_{1}$ | ${df}_{2}$ | $p$ |
| --- | --- | --- | --- | --- | --- | --- |
| Participant group | .05 | [.02, .09] | 19.97 | 1 | 399 | < .001 |
| Gender | .00 | [.00, .01] | 0.25 | 1 | 399 | .615 |
| Age | .04 | [.01, .08] | 6.01 | 3 | 399 | < .001 |
| Target group | .05 | [.02, .09] | 21.56 | 1 | 399 | < .001 |
| Participant group $\times$ Target group | .01 | [.00, .03] | 2.55 | 1 | 399 | .111 |
| Gender $\times$ Target Group | .03 | [.01, .06] | 12.88 | 1 | 399 | < .001 |
| Age $\times$ TargetGroup | .00 | [.00, .01] | 0.64 | 3 | 399 | .592 |

**First-person shooter task.** We observed shooter biases in reactions times and error rates for both, police and civilian participants after controlling for age and gender. (Table S8).

**Mediation model: Social dominance orientation and negative contact during the job as mediators for perceived trustworthiness of Arab individuals controlling for age and gender.** A mediation model was estimated in which Participant group predicted trust in Arab targets directly and indirectly through social dominance orientation (SDO) and negative job‑related contact, controlling for perceived trustworthiness for White targets, age, and gender. The direct effect of Participant group on trust in Arab targets was significant, *b* = –0.19, *SE* = 0.09, *z* = –2.11, *p* = .035. Sample also significantly predicted both mediators (SDO: *b* = 0.28, p = .002; negative contact: b = 1.89, *p* < .001). Both indirect effects were statistically significant based on 1,000 bootstrap samples: through SDO, indirect effect = –0.07, 95% CI [–0.11, –0.02], and through negative contact, indirect effect = –0.10, 95% CI [–0.21, –0.01]. The total effect of Participant group on perceived trustworthiness for Arab targets was significant, *b* = –0.35, *SE* = 0.08, *z* = –4.26, *p* < .001. These results indicate that differences between samples in trust toward Arab targets were still (partially) mediated by SDO and negative job‑related contact when controlling for participant age and gender.

**Table S8**
*ANOVA results for reaction times and errors in the first-person shooter task controlling for participant age group and gender*

|  | Reaction times | | | | | | Error rates | | | | | |
| --- | --- | --- | --- | --- | --- | --- | --- | --- | --- | --- | --- | --- |
| Effect | $\hat{\eta}_{p}^{2}$ | 90% CI | $F$ | ${df}_{1}$ | ${df}_{2}$ | $p$ | $\hat{\eta}_{p}^{2}$ | 90% CI | $F$ | ${df}_{1}$ | ${df}_{2}$ | $p$ |
| PG | .00 | [.00, .01] | 0.19 | 1 | 410 | .665 | .07 | [.03, .11] | 29.21 | 1 | 412 | < .001 |
| Gender | .00 | [.00, .02] | 0.94 | 1 | 410 | .333 | .02 | [.00, .05] | 7.86 | 1 | 412 | .005 |
| Age | .06 | [.03, .10] | 9.31 | 3 | 410 | < .001 | .01 | [.00, .03] | 1.81 | 3 | 412 | .145 |
| OT | .42 | [.36, .47] | 290.95 | 1 | 410 | < .001 | .01 | [.00, .03] | 4.14 | 1 | 412 | .043 |
| TG | .07 | [.04, .12] | 33.18 | 1 | 410 | < .001 | .01 | [.00, .04] | 6.10 | 1 | 412 | .014 |
| PG $\times$ OT | .01 | [.00, .04] | 5.29 | 1 | 410 | .022 | .00 | [.00, .01] | 0.05 | 1 | 412 | .817 |
| Gender $\times$ OT | .01 | [.00, .02] | 2.33 | 1 | 410 | .128 | .00 | [.00, .02] | 1.33 | 1 | 412 | .250 |
| Age $\times$ OT | .02 | [.00, .04] | 2.94 | 3 | 410 | .033 | .00 | [.00, .01] | 0.23 | 3 | 412 | .875 |
| PG $\times$ TG | .00 | [.00, .01] | 0.07 | 1 | 410 | .792 | .00 | [.00, .01] | 0.12 | 1 | 412 | .729 |
| Gender $\times$ TG | .00 | [.00, .00] | 0.03 | 1 | 410 | .873 | .00 | [.00, .00] | 0.02 | 1 | 412 | .899 |
| Age $\times$ TG | .00 | [.00, .00] | 0.11 | 3 | 410 | .954 | .01 | [.00, .02] | 1.32 | 3 | 412 | .268 |
| OT $\times$ TG | .04 | [.01, .07] | 13.11 | 1 | 410 | < .001 | .05 | [.02, .09] | 17.59 | 1 | 412 | < .001 |
| PG $\times$ OT $\times$ TG | .00 | [.00, .01] | 0.04 | 1 | 410 | .847 | .00 | [.00, .02] | 0.77 | 1 | 412 | .380 |
| Gender $\times$ OT $\times$ TG | .00 | [.00, .01] | 0.33 | 1 | 410 | .564 | .00 | [.00, .01] | 0.46 | 1 | 412 | .499 |
| Age $\times$ OT $\times$ TG | .01 | [.00, .03] | 1.20 | 3 | 410 | .308 | .01 | [.00, .02] | 0.84 | 3 | 412 | .472 |

*Note.* PG = Participant Group, OT = Object Type, TG = Target Group
